# Bibliography

**Published:** 1894-06

**Authors:** 


### Bibliography.

CATCHING'S COMPENDIUM OF PRACTICAL DENTISTRY FOR 1893. B. H. Catching, D.D.S., Editor and Publisher, Atlanta, Georgia.

This work, just issued, embraces a very complete record of the valuable material of our journalistic literature for the year 1893, and, as a work of reference for the busy dentist, is of very great value.

It embraces not only the matter of the journals in the English language, but also that of those of other languages as well, which constitutes a very valuable addition to the work.

The preparation of such a work involves a great amount of labor in reading and collation. Such a volume is a very great addition to the library of the dentist, and should be in the hands of every dentist who desires to have most ready access to the valuable matter contained in our journals. We cannot too highly commend this work to the attention of the profession.

